# Platelet inhibition during ticagrelor monotherapy versus ticagrelor plus aspirin in patients with coronary artery disease (TEMPLATE study): study protocol for a randomised controlled trial

**DOI:** 10.1186/s13063-017-2277-9

**Published:** 2017-11-09

**Authors:** Sarah Baos, Wendy Underwood, Lucy Culliford, Barnaby C. Reeves, Chris A. Rogers, Ruth Bowles, Tom Johnson, Andreas Baumbach, Andrew Mumford

**Affiliations:** 1 Clinical Trials and Evaluation Unit, School of Clinical Sciences, University of Bristol, Bristol, UK; 2 Bristol Heart Institute, University Hospitals Bristol, Bristol, UK; 3 School of Cellular and Molecular Medicine, University of Bristol, Bristol, UK

**Keywords:** Antiplatelet therapy, Platelet function, Haematology, Ticagrelor, Clopidogrel, Prasugrel, Aspirin, P2Y_12_ blocker

## Abstract

**Background:**

Dual antiplatelet therapy (DAPT) with aspirin (ASP) and a P2Y_12_ blocker is currently standard care after percutaneous coronary intervention (PCI) with stent insertion, and aims to inhibit platelet function in order to prevent stent thrombosis. The P2Y_12_ blocker ticagrelor (TIC) has greater antiplatelet effect than the previously used members of this class, such as clopidogrel. In healthy volunteers, TIC is sufficient to cause strong platelet inhibition, with little additional effect from ASP. Omission of ASP may improve the safety of antiplatelet regimes by reducing bleeding. However, the effect of single antiplatelet treatment with TIC, compared to DAPT with TIC + ASP, has not been studied in detail in patients with coronary artery disease.

**Methods:**

To compare TIC with TIC + ASP, we have initiated a single centre, open-label randomised controlled trial (TEMPLATE study) in adults receiving DAPT following PCI with a sample size of 110 patients. Patients are invited to join the study when, as part of standard care, they are due to switch from DAPT (ASP + any P2Y_12_ blocker) to single antiplatelet treatment with ASP alone after 6–12 months. Patients are randomised to receive either TIC or TIC + ASP for 4 weeks. All patients then revert to standard care with ASP alone. Blood samples and clinical data are collected at three study visits: at baseline during treatment with ASP + any P2Y_12_ blocker (visit 1); approximately 4 weeks after visit 1 during treatment with either TIC or TIC + ASP (visit 2); and approximately 8 weeks after visit 1 when treatment has reverted to ASP alone (visit 3). The primary outcome is the extent of platelet inhibition, measured by light transmission aggregation, flow cytometry, flow chamber and plasma biomarker tests. The primary analysis will compare the extent of platelet inhibition between the TIC and TIC + ASP groups at visit 2, adjusted for baseline platelet reactivity. Secondary analyses will compare the extent of platelet inhibition at visit 2 with that at visit 3.

**Discussion:**

This is the first study to compare in detail the extent of platelet inhibition in patients who are receiving TIC compared with TIC + ASP. The study findings will complement larger-scale trials of the clinical efficacy and safety of TIC compared to TIC + ASP.

**Trial registration:**

ISRCTN registry, identifier ISRCTN84335288. Registered on 23 June 2014.

**Electronic supplementary material:**

The online version of this article (doi:10.1186/s13063-017-2277-9) contains supplementary material, which is available to authorized users.

## Background

Dual antiplatelet therapy (DAPT) with aspirin (ASP) and a P2Y purinoreceptor (P2Y_12_) blocker is standard care for patients with coronary artery disease in several settings, including after acute coronary syndrome (ACS) and percutaneous coronary intervention (PCI) with or without coronary stent insertion [1–3]. The rationale for this treatment is that most adverse cardiac events that occur in these settings result from the formation of abnormal platelet aggregates causing coronary artery or stent thrombosis. Inhibition of platelets by simultaneously targeting both the thromboxane A_2_ pathway (with ASP) and the P2Y_12_ receptor pathway (with a P2Y_12_ blocker) results in strong platelet inhibition and helps prevent aggregate formation [2]. The intensity of antiplatelet treatment is typically reduced at 6–12 months following ACS or PCI by switching patients from DAPT to single-agent treatment with ASP alone. This ensures strong platelet inhibition in the highest risk period after ACS or PCI and minimises the long-term bleeding risk associated with DAPT.

When used in combination with ASP, clopidogrel (CLOP; the first widely used P2Y_12_ blocker) reduced the incidence of major adverse cardiac events compared to single antiplatelet treatment with ASP [4]. However, approximately 30% of individuals display incomplete inhibition of the platelet P2Y_12_ receptor pathway with CLOP, resulting in reduced efficacy [5]. These limitations have been circumvented by the development of strong P2Y_12_ blockers such as ticagrelor (TIC), which give greater and more consistent inhibition of the P2Y_12_ receptor pathway compared with CLOP. Accordingly, TIC in combination with ASP is associated with fewer major adverse cardiac events than CLOP in combination with ASP [6].

More recent observations made in experimental models of platelet inhibition [7] and in observational studies of healthy volunteers receiving antiplatelet treatments [4] suggest that strong P2Y_12_ blockers such as TIC used alone result in a consistent and full platelet inhibition, and that little further platelet inhibition is achieved by the addition of ASP. In response to these findings, it has been proposed that medication with strong P2Y_12_ blockers without additional ASP may be sufficient to achieve full therapeutic antiplatelet effect in clinical settings in which DAPT is currently standard care [8]. Avoidance of ASP may also confer safety benefits because ASP is associated with gastrointestinal bleeding due to its local effect on gastric mucosa, unrelated to the antiplatelet effect [9].

Single antiplatelet treatment with TIC is currently being evaluated in the GLOBAL LEADERS study, which is a large, international, multicentre, randomised controlled trial of TIC versus TIC + ASP following PCI and coronary stent insertion [10]. However, the main endpoints of this trial are all-cause mortality or non-fatal myocardial infarction and bleeding. The GLOBAL LEADERS study does not include a detailed laboratory evaluation of the pharmacodynamic effect of the two different treatment regimes on platelet inhibition.

## Aims and objectives

The main aim of the TEMPLATE study is to determine the extent of platelet inhibition in patients with coronary artery disease achieved with single antiplatelet treatment with TIC compared with DAPT with TIC + ASP. The secondary aim is to compare the effect of TIC or TIC + ASP to single antiplatelet treatment with ASP alone.

The study objectives are to perform laboratory measurements of platelet inhibition in patients at the following points of treatment with antiplatelet drugs after PCI: (i) at the end of DAPT with ASP + any P2Y_12_ blocker treatment; (ii) after 4 weeks of treatment with either TIC or TIC + ASP; and, (iii) after all patients are switched to ASP alone after the study intervention.

## Methods

### Study design and setting

The TEMPLATE study is a single centre, open-label, randomised controlled trial of the effects of different antiplatelet treatments on platelet laboratory test results in patients who have undergone PCI and coronary stent insertion at the Bristol Heart Institute (BHI).

### Study population

The study population is patients at the BHI who have been prescribed DAPT as part of standard clinical care following PCI and coronary stent insertion.

A patient will be eligible for inclusion if: (i) prescribed DAPT with ASP + any P2Y_12_ blocker for a minimum interval of 4 weeks; and (ii) intending to transfer to ASP after cessation of treatment with ASP + any P2Y_12_ blocker; and, (iii) aged over 18 years at the point of randomisation.

Patients will be ineligible if any of the following apply: (i) contraindication to DAPT; (ii) treatment with ASP + any P2Y_12_ blocker after PCI was interrupted or terminated because of bleeding or increased bleeding risk; (iii) contraindication to TIC; (iv) pregnancy and or breast feeding; (v) the patient is a woman of childbearing potential who is unwilling to use contraception; (vi) the patient is a man with a spouse or partner with childbearing potential unless the patient is sterilised or has agreed to use barrier contraceptives.

Concomitant medications which may modify the therapeutic effect of TIC or which may have their therapeutic effect modified by TIC are not absolute exclusions to the study, but will be considered as potential reasons for exclusion by the enrolling clinician. These include: (i) strong CYP3A4 inducers (e.g. rifampicin, dexamethasone, phenytoin, carbamazepine and phenobarbital), (ii) CYP3A4 substrates with narrow therapeutic indices (e.g. cisapride, ergot alkaloids, simvastatin or lovastatin, (iii) digoxin, and (iv) potent P-glycoprotein inhibitors (e.g. verapamil, quinidine, cyclosporin).

Concomitant medications with a potential adverse interaction when co-prescribed with ASP will be considered as potential reasons for exclusion by the enrolling clinician. These include: some diuretics, probenecid, heparin, phenindione, antiepileptics such as phenytoin, warfarin, insulin and sulphonylurea hypoglycaemic agents, non-steroidal anti-inflammatory drugs, methotrexate, corticosteroids, antacids, iron salts, carbonates, alkali hydroxides and ibuprofen.

### Patient approach and study visits

**Fig. 1:**
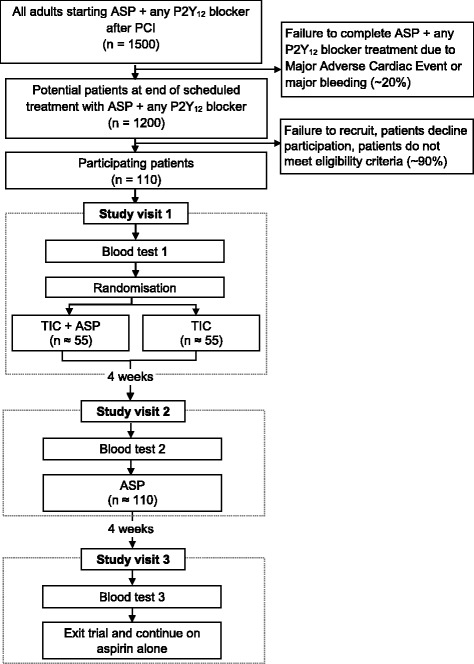
Trial schema. Schema showing the recruitment pathway with the number of patients to be recruited, anticipated eligibility, recruitment and follow-up rates. *ASP* aspirin, *CLOP* clopidogrel, *TIC* ticagrelor

Potentially eligible patients will be identified using hospital databases which record the demographic details of patients who undergo interventional cardiology procedures at the BHI and the antiplatelet treatment prescribed after the procedure. The first approach will be an invitation letter and patient information leaflet. Since most patients in the study population were receiving DAPT for 6–12 months, this was usually sent by post approximately 6 weeks before the planned completion of DAPT. Potentially eligible patients who reply expressing an interest in taking part will be contacted by telephone and invited to attend three outpatient study visits at the BHI. They will be instructed to continue with their existing DAPT until the first study visit. A study flow diagram is shown in Fig. 1.

**Fig. 2:**
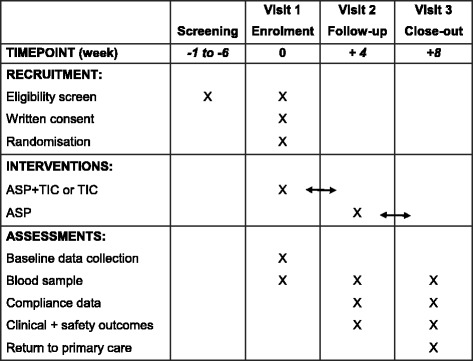
Schedule of data collection (SPIRIT)

At each of three study visits, clinical data will be collected and an 18 mL venous blood sample will be obtained by peripheral venepuncture for laboratory analysis. The schedule of data collection is shown in Fig. 2. All data will be collected on a bespoke database and stored on a secure server.

## Study visit 1

Study visit 1 will occur when a patient would be switching from DAPT with ASP + any P2Y_12_ blocker to ASP alone in the standard clinical care pathway. A member of the research team will describe the study, answer questions and confirm eligibility. Eligible patients will be invited to complete and sign the study consent form.

### Randomisation

Allocation to the TIC + ASP group or the TIC group in a 1:1 ratio will be performed by an authorised member of the research team using a secure internet-based randomisation system (http://www.sealedenvelope.com/), using block randomisation with blocks of varying sizes. Clinicians, research nurses, trial coordinators, pharmacists and patients will be aware of the allocation. As the trial is open label, no code-breaking procedure is required. Since the costs associated with production of a placebo medication were prohibitively high, we adopted a pragmatic open-label design in which the study patients, their clinicians, research nurse and trials coordinator will not be blinded to treatment allocation. We do not anticipate this to have a significant impact on trial results. The laboratory staff will be blinded to group allocation.

### Trial interventions

Patients randomised to the TIC + ASP group will transfer from ASP + any P2Y_12_ blocker to ASP 75 mg once per day plus a loading dose of TIC 180 mg followed by TIC 90 mg twice per day. Patients randomised to the TIC group will transfer from ASP + any P2Y_12_ blocker to a loading dose of TIC 180 mg followed by TIC 90 mg twice per day. The study medication will continue to be taken until study visit 2 at 28 days (acceptable range 21–35 days) after enrolment and randomisation. The study medication will be stored and dispensed by the trial site’s pharmacy in accordance with Good Clinical Practice. Patients will be asked to record any missed doses, and to return any unused medication at their second study visit to ascertain adherence. Patients will be instructed to take the next scheduled dose as usual, if they miss a dose.

## Study visit 2

Patients will be interviewed by the study team to collect safety data and to check compliance with the study medication (Fig. 2). The safety data will include adverse events potentially associated with the study medication, the occurrence of major adverse cardiac events and bleeding, categorised using the Bleeding Academic Research Consortium (BARC) definitions of bleeding (Table 1) [11].

**Table 1:** Bleeding Academic Research Consortium (BARC) definitions

Class	Definition
Type 0	No bleeding.
Type 1	Bleeding that is not actionable and does not cause the patient to seek unscheduled performance of studies, hospitalisation, or treatment by a healthcare professional; may include episodes leading to self-discontinuation of medical therapy by the patient without consulting a healthcare professional.
Type 2	Any overt, actionable sign of haemorrhage (e.g. more bleeding than would be expected for a clinical circumstance, including bleeding found by imaging alone) that does not fit the criteria for type 3, 4, or 5 but does meet at least one of the following criteria: requiring nonsurgical, medical intervention by a healthcare professional, leading to hospitalisation or increased level of care, prompting evaluation.
Type 3a	Overt bleeding plus haemoglobin drop of 3 to < 5 g/dL, corrected for transfusion (provided haemoglobin drop is related to bleed) OR any transfusion with overt bleeding.
Type 3b	Overt bleeding plus haemoglobin drop < 5 g/dL, corrected for transfusion (provided haemoglobin drop is related to bleed) OR cardiac tamponade. Bleeding requiring surgical intervention for control (excluding dental/nasal/skin/haemorrhoid) OR bleeding requiring intravenous vasoactive agents.
Type 3c	Intracranial haemorrhage (does not include microbleeds or haemorrhagic transformation, does include intraspinal) Subcategories confirmed by autopsy or imaging or lumbar puncture OR intraocular bleed compromising vision.
Type 4	Coronary artery bypass graft (CABG)-related bleeding OR perioperative intracranial bleeding within 48 h OR reoperation after closure of sternotomy for the purpose of controlling bleeding OR transfusion of ≥ 5 U whole blood or packed red blood cells within a 48 h period (cell saver product are not included) OR chest tube output ≥ 2 L within a 24-h period.*
Type 5a	Probable fatal bleeding; no autopsy or imaging confirmation but clinically suspicious.
Type 5b	Definite fatal bleeding; overt bleeding or autopsy or imaging confirmation

*If a CABG-related bleed is not adjudicated as at least a type 3 severity event, it will be classified as not a bleeding event. If a bleeding event occurs with a clear temporal relationship to CABG (i.e. within a 48-h time frame) but does not meet type 4 severity criteria, it will be classified as not a bleeding event

All patients will then be transferred to ASP 75 mg once per day, thereby returning to standard clinical care. This treatment will continue until study visit 3 which will be 28 days (acceptable range 21–35 days) after study visit 2, and thereafter, long-term. Patients who are unable to attend study visit 2 within 35 days from study visit 1 will be advised to transfer to ASP 75 mg once per day at 35 days after study visit 1 and will be invited to attend for a final study visit at the same time as their planned study visit 3.

## Study visit 3

Patients will be interviewed by the study team to collect safety data and to check compliance with the prescribed medication. Patients will then exit the study and will be instructed to continue ASP 75 mg once per day as per standard clinical care under the ongoing supervision of their primary care practitioner (Fig. 2).

### Definition of end of trial

Patients will end their involvement in the study after they have completed study visit 3, at approximately 8 weeks after study visit 1. The end of the study is when analyses of blood samples are completed, data queries are resolved, the database is locked and the analysis completed.

### Loss to follow-up and withdrawals

Each patient has the right to withdraw at any time. In this event, we will analyse any data already collected, unless the patient expresses a wish for their samples to be destroyed and any associated data not to be included in the analysis. We expect low rates of patient withdrawal and losses to follow-up because the study interval is only approximately 8 weeks and requires only three study visits. It is also unlikely that patients will have to be withdrawn from their allocated study treatment as the study intervention (TIC + ASP or TIC) is well tolerated and is prescribed for only a 4-week period. However, in the event of a serious adverse event that the investigator believes may be attributable to the intervention, the study medication will be discontinued and the patient will be returned to standard care with ASP 75 mg once daily. The trial medication may also be discontinued if any contraindicated therapy is initiated whilst the patient is participating in the trial. Data collected before discontinuation of trial medication will be included in the analyses.

### Laboratory analyses

The blood samples obtained at the three study visits will be analysed using a panel of platelet function and biomarker tests in a central laboratory. All laboratory tests will be performed in accordance with reagent manufacturers’ instructions and with locally generated standard operating procedures. The following analyses will be performed:

(i) Light transmission aggregation (LTA) in platelet-rich plasma (PRP) in response to stimulation with collagen-related peptide (CRP), adenosine diphosphate (ADP), arachidonic acid (AA), thromboxane receptor agonist (U46619) and thrombin-related activator peptide 6 (TRAP 6).
(ii) Flow cytometry to quantify surface CD62P expression and PAC-1 binding in unstimulated platelets and in platelets stimulated with CRP and TRAP 6.
(iii) Adhesion and aggregation of platelets in whole blood on a collagen surface under flow conditions.
(iv) Plasma concentrations of soluble CD40 ligand (sCD40L) and thromboxane B2 (TXB2) to assess baseline in vivo platelet activation and thromboxane A2 biosynthesis respectively. These biomarkers will be measured by enzyme-linked immunosorbent assay using frozen plasma samples, after the end of recruitment.

### Primary outcome

The primary outcome is the maximum amplitude of the LTA response to TRAP 6 expressed as a percentage of the absolute difference in light transmission between PRP and platelet-poor plasma. This was selected as the primary outcome since the functional response to TRAP 6 represents the best marker of the overall extent of platelet inhibition.

### Secondary outcomes

The secondary outcomes will be the following laboratory test results:

(i) Maximum amplitude of the LTA responses to CRP, ADP, AA and U46619.
(ii) Median fluorescence intensity of anti-P selectin and PAC-1 binding to platelets in unstimulated platelets and after stimulation with CRP and with TRAP 6.
(iii) Aggregation and adhesion parameters of platelets exposed to a collagen surface under flow conditions.
(iv) Plasma concentrations of sCD40L and TXB2.

### Sample size

The sample size has been set at 110 patients, which is sufficient to detect a standardised difference in the primary outcome between the two study interventions of 0.35 standard deviations. The sample size has been calculated with the following assumptions:

1. Correlation between the primary outcome measured at visit 1 and at visit 2 = 0.5
2. No cross-over anticipated
3. 10% loss to follow-up (i.e. missing outcome data at visit 2)

DAPT with ASP + any P2Y_12_ blocker is prescribed as part of standard current care at the BHI to approximately 1500 adults per year who undergo PCI and coronary stent insertion. We estimate that approximately 1200 (80%) will complete the prescribed duration of treatment of 6–12 months. Based on previous observational studies at the BHI, we conservatively estimate that the overall recruitment and completion rate will be approximately 10% of eligible patients. Therefore, we predict that 110 patients will occur in 14–16 months.

### Statistical analysis

The laboratory test results will be expressed as continuous variables. The results for the primary comparison of the maximum amplitude of the LTA response to TRAP 6 between the TIC + ASP and TIC groups will be analysed using linear mixed regression. The baseline (visit 1) and post-intervention values (visit 2) will be modelled jointly in preference to the pre-randomisation value being modelled as a covariate to avoid the necessity either to exclude cases with missing pre-intervention measures or to impute missing pre-intervention values. The secondary outcome measures will be compared similarly.

The study will also enable the extent of platelet inhibition to be described within subjects over time as their treatment changes (i.e. from visit 1 to visit 2 to visit 3).

Comparative analyses will be on the basis of intention to treat and mean differences will be quantified with 95% confidence intervals. The width of the individual confidence intervals for the secondary outcomes will be adjusted for the number of tests carried out (Bonferroni correction).

The primary analysis will take place when follow-up and laboratory analyses are complete for all recruited patients. No interim or subgroup analyses are planned other than those outlined above. Safety data will be reviewed regularly by the trial management group who meet at frequent intervals to discuss trial progress. During these meetings the rate of adverse events in the study population will be monitored against the anticipated rate of events that are expected for the target population.

### Research approval

Research ethics approval and clinical trial authorisation were granted in October 2014 by the South-Central Oxford A Research Ethics Committee (reference 14/SC/1309) and the Medicines and Healthcare Regulatory Agency (EUDRACT No. 2013-002734-20) respectively. The trial is managed by the Clinical Trials and Evaluation Unit Bristol (CTEU Bristol) and sponsored by the University Hospitals Bristol NHS Foundation Trust (www.uhbristol.nhs.uk/research-innovation/). Trial governance is through the sponsor’s standard operating procedures. The trial is registered as ISRCTN84335288 and conforms to SPIRIT guidelines (see Fig. 2 and Additional file 1: SPIRIT checklist).

### Changes to the protocol since first approved

There have been three substantial amendments made to the protocol since it was first approved. The first amendment was approved in May 2015 and incorporated minor clarifications in addition to the new recommendations from the European Society of Cardiologists. These recommendations led to a change in duration of standard DAPT specified in the protocol, from 12 months to between 6 and 12 months after PCI and insertion of a drug-eluting stent; this change increased the pool of eligible patients.

The second amendment to the protocol was approved in August 2015, removing the plan to establish a Data Monitoring and Safety Committee (DMSC). This change was made because, on review of the study design, it became apparent that the study would not benefit from a DMSC. Instead, the safety data are being reviewed closely by the trial management group and the Steering Committee for Cardiovascular Studies, a subgroup of the University Hospitals Bristol NHS Foundation Trust Cardiovascular Research Board.

The third amendment was approved in September 2016. This amendment widened the inclusion criteria from receiving ASP + CLOP for 6–12 months to receiving ASP + any P2Y_12_ blocker for 6–12 months. This widening of the inclusion criteria was made to enhance study enrolment because shortly after starting the study, the institutional standard care changed from using CLOP as the preferred P2Y_12_ blocker, to other P2Y_12_ blockers for some patient subgroups. After this amendment, the enrolment rate increased to that predicted in the original study design.

## Discussion

The TEMPLATE study design will enable unbiased comparison of the effects of TIC versus TIC + ASP on platelet activity in patients with coronary artery disease. It will also enable a longitudinal comparison of the effects TIC and TIC + ASP with the effects of ASP alone in the same patients. The laboratory tests selected for this study will enable measurement of functional platelet responses to a panel of activating agonists using LTA, flow cytometry and flow chamber tests, selected to measure the extent of inhibition of the multiple platelet activation pathways. We will also measure the extent of baseline platelet activity by testing unstimulated platelets by flow cytometry and with the soluble platelet activation biomarker tests. Together, these data will provide a comprehensive description of the overall pharmacodynamic effects of the different antiplatelet treatments. This information has not been reported previously in cohorts of patients with coronary artery disease receiving TIC or TIC + ASP. To our knowledge, there are no similar previous studies in this patient group that have evaluated the pharmacodynamic effect of monotherapy with other P2Y_12_ blockers, including CLOP.

The TEMPLATE study design allows the information to be obtained in a low-risk setting. We are enrolling patients at the point in the standard clinical care pathway when there is a transition from DAPT to single-antiplatelet treatment with ASP, typically at 6 to 12 months after the PCI procedure. This is after the interval of greatest risk of major adverse cardiac events and abnormal bleeding following PCI [12]. Furthermore, the duration of exposure to the study treatment (i.e. either TIC or TIC + ASP) is only 4 weeks, which will minimise the likelihood of adverse events attributable to the study drugs. The short overall study participation of 8 weeks during which there are only three study visits should facilitate study delivery and minimise patient drop-out.

The main benefit of the TEMPLATE study is that the findings will provide evidence to either support or refute the hypothesis that TIC monotherapy enables platelet inhibition to the same extent as dual therapy with TIC + ASP. This is significant clinically because abnormal bleeding is a major complication of all antiplatelet therapy, but particularly of ASP which adversely affects gastric mucosa, increasing the risk of gastrointestinal bleeding [9]. If the findings of the TEMPLATE study indicate no difference in platelet inhibition between the TIC treatment group and the TIC + ASP treatment group, then this would support avoidance of ASP in this patient group, potentially conferring a safety advantage. This is also part of the rationale of the large-scale GLOBAL LEADERS randomised controlled trail (NCT01813435) of 16,000 participants in which the study group receive TIC + ASP for 1 month after PCI and coronary stent insertion followed by TIC monotherapy for a further 23 months. The control group is DAPT with TIC + ASP or CLOP + ASP for 12 months [10]. The outcomes of the GLOBAL LEADERS study are all-cause mortality or non-fatal new Q-wave myocardial infarction (composite primary outcome) and bleeding (secondary outcome), but do not include pharmacodynamic measures of the effect of the different antiplatelet regimes. We anticipate that reporting of TEMPLATE study results in a peer-reviewed publication will precede reporting of the GLOBAL LEADERS study results and that our results will assist interpretation and help inform future treatment guidelines.

### Study status

The trial opened to recruitment in December 2015 with the first patient recruited in January 2016. Recruitment is ongoing.

## Trial steering committee members

The Trials Steering Committee comprises a consultant anaesthetist, interventional consultant cardiologist and a consultant cardiac surgeon.

## Additional file

Additional file 1: SPIRIT 2013 checklist: recommended items to address in a clinical trial protocol and related documents. (DOC 123 kb)

## Abbreviations

AA: Arachidonic acid (a platelet metabolite that stimulates platelets in vivo and is also used as a laboratory reagent to study platelet activation responses)
ACS: Acute coronary syndrome
ADP: Adenosine diphosphate (a P2Y_12_ receptor agonist that stimulates platelets in vivo and is also used as a laboratory reagent to study platelet activation responses)
ASP: Aspirin
BARC: Bleeding Academic Research Consortium
BHI: Bristol Heart Institute
CABG: Coronary artery bypass grafting
CLOP: Clopidogrel
CTEU Bristol: Clinical Trials and Evaluation Unit Bristol
CRP: Collagen-related peptide (a platelet activating chemical that is used as a laboratory reagent to study platelet activation responses)
DAPT: Dual antiplatelet therapy
DMSC: Data monitoring and safety committee
LTA: Light transmission aggregation
NHS: National Health Service
NIHR: National Institute for Health Research
P2Y_12_: P2Y purinoreceptor 12
PCI: Percutaneous coronary intervention
PRP: Platelet-rich plasma
sCD40L: soluble CD40 ligand (a soluble plasma biomarker of platelet activation in vivo)
TXB2: Thromboxane B2 (a soluble plasma biomarker of platelet activation)
TIC: Ticagrelor
TRAP 6: Thrombin receptor agonist peptide-6 (a platelet PAR1 receptor agonist that is used as a laboratory reagent to study platelet activation responses)
U46619: A thromboxane receptor agonist that is used as a laboratory reagent to study platelet activation responses

## References

[1] Antithrombotic Trialists Collaboration (2002). Collaborative meta-analysis of randomised trials of antiplatelet therapy for prevention of death, myocardial infarction, and stroke in high risk patients. BMJ..

[2] Mehta SR (2001). Effects of pretreatment with clopidogrel and aspirin followed by long-term therapy in patients undergoing percutaneous coronary intervention: the PCI-CURE study. Lancet.

[3] Anderson JL (2011). 2011 ACCF/AHA focused update incorporated into the ACC/AHA 2007 guidelines for the management of patients with unstable angina/non-ST-elevation myocardial infarction: a report of the American College of Cardiology Foundation/American Heart Association Task Force on Practice Guidelines. Circulation.

[4] Leadbeater PDM (2011). Aspirin has little additional anti-platelet effect in healthy volunteers receiving prasugrel. J Thromb Haemost.

[5] Sibbing D (2010). Response to letter regarding article, “Cytochrome 2C19*17 allelic variant, platelet aggregation, bleeding events, and stent thrombosis in clopidogrel-treated patients with coronary stent placement”. Circulation.

[6] Wallentin L (2009). Ticagrelor versus clopidogrel in patients with acute coronary syndromes. New Engl J Med.

[7] Armstrong PCJ (2010). Reduction of platelet thromboxane A(2) production ex vivo and in vivo by clopidogrel therapy. J Thromb Haemost.

[8] Warner TD (2010). Dual antiplatelet therapy in cardiovascular disease: does aspirin increase clinical risk in the presence of potent P2Y(12) receptor antagonists?. Heart.

[9] FitzGerald GA (1983). Endogenous biosynthesis of prostacyclin and thromboxane and platelet function during chronic administration of aspirin in man. J Clin Invest.

[10] Vranckx P (2016). Long-term ticagrelor monotherapy versus standard dual antiplatelet therapy followed by aspirin monotherapy in patients undergoing biolimus-eluting stent implantation: rationale and design of the GLOBAL LEADERS trial. EuroIntervention.

[11] Mehran R (2011). Standardized bleeding definitions for cardiovascular clinical trials a consensus report from the bleeding academic research consortium. Circulation.

[12] Pandit A (2015). Shorter (</=6 months) versus longer (>/=12 months) duration dual antiplatelet therapy after drug eluting stents: a meta-analysis of randomized clinical trials. Catheter Cardiovasc Interv.

